# Recurrent Deep (Aggressive) Angiomyxoma of the Pelvis: Serial MRI Documentation of Sustained Complete Radiologic Response During Eight Years of Gonadotropin-Releasing Hormone (GnRH) Agonist Therapy

**DOI:** 10.7759/cureus.114829

**Published:** 2026-08-20

**Authors:** Beatriz Fevereiro, Ricardo Fonseca, Teresa Margarida Cunha

**Affiliations:** 1 Radiology Department, Unidade Local de Saúde de Matosinhos, Porto, PRT; 2 Pathology Department, Instituto Português de Oncologia de Lisboa Francisco Gentil, Lisboa, PRT; 3 Radiology Department, Instituto Português de Oncologia de Lisboa Francisco Gentil, Lisboa, PRT

**Keywords:** aggressive angiomyxoma, gnrh agonist, hormonal therapy, mesenchymal tumor, mri, pelvic tumor, perineal soft tissue mass

## Abstract

Deep (aggressive) angiomyxoma is a rare mesenchymal tumor that primarily affects premenopausal women and is frequently misdiagnosed because of its deep pelvic location, indolent growth, and nonspecific clinical presentation. It is typically multicompartmental and demonstrates a characteristic laminated (“swirled”) appearance on T2-weighted magnetic resonance imaging (MRI), reflecting alternating myxoid and fibrous stromal components. Local recurrence is common after surgical resection, making long-term imaging surveillance essential. Tumor expression of estrogen and progesterone receptors provides a biological rationale for hormonal therapy.

We report a case of recurrent pelvic deep (aggressive) angiomyxoma in a 45-year-old woman who achieved complete and sustained radiologic remission documented by serial MRI throughout 8 years of continuous gonadotropin-releasing hormone (GnRH) agonist therapy. This uncommon long-term outcome highlights both the potential for durable disease control with hormonal therapy in deep (aggressive) angiomyxoma and the value of MRI for longitudinal assessment of treatment response.

## Introduction

Deep (aggressive) angiomyxoma (AAM) is a rare, benign, myxoid spindle-cell mesenchymal tumor that predominantly affects premenopausal women and typically arises from the deep soft tissues of the vulvo-perineal region [1-4]. Less commonly, it occurs in analogous locations in men or at unusual sites, including the lung, liver, kidney, larynx, and orbit [3,5].

Patients may present with pelvic or perineal discomfort or symptoms related to mass effect [6]. Physical examination frequently underestimates tumor extent because the deep pelvic component may be clinically occult. Consequently, AAM may be mistaken for more common conditions, including Bartholin or vaginal cysts, lipomas, leiomyomas, rectoceles, and perineal hernias [1,2,5].

Despite its benign histology, AAM exhibits slow but locally infiltrative growth, making complete surgical excision challenging. The term “aggressive” reflects its propensity for local recurrence, even after complete resection. Recurrence rates of up to 70% have been reported, usually within the first few years after surgery, although late recurrences have also been described, emphasizing the importance of long-term surveillance [3,5,7,8].

The frequent expression of estrogen and progesterone receptors in AAM provides a biological rationale for hormonal therapy, which has emerged as an important treatment option in selected patients [5,7-10]. However, evidence regarding its long-term efficacy and optimal treatment duration remains limited. Sustained complete radiologic responses documented by serial imaging over prolonged follow-up are also rarely reported [9,10].

We present a case of recurrent pelvic AAM with radiopathological correlation and serial MRI documentation of a complete radiologic response sustained throughout eight years of continuous gonadotropin-releasing hormone (GnRH) agonist therapy.

## Case presentation

A 45-year-old premenopausal woman presented with a slowly enlarging right perineal mass of several months' duration, associated with difficulty with defecation. Physical examination revealed an approximately 6 cm, mobile, non-tender lesion in the right perineal region. Her medical history was notable for a hysterectomy for multiple uterine leiomyomas, with preservation of both ovaries. There was no relevant family history. No other symptoms were reported, including constitutional, urinary, or hormone-related symptoms. Laboratory tests were unremarkable.

**Figure 1:**
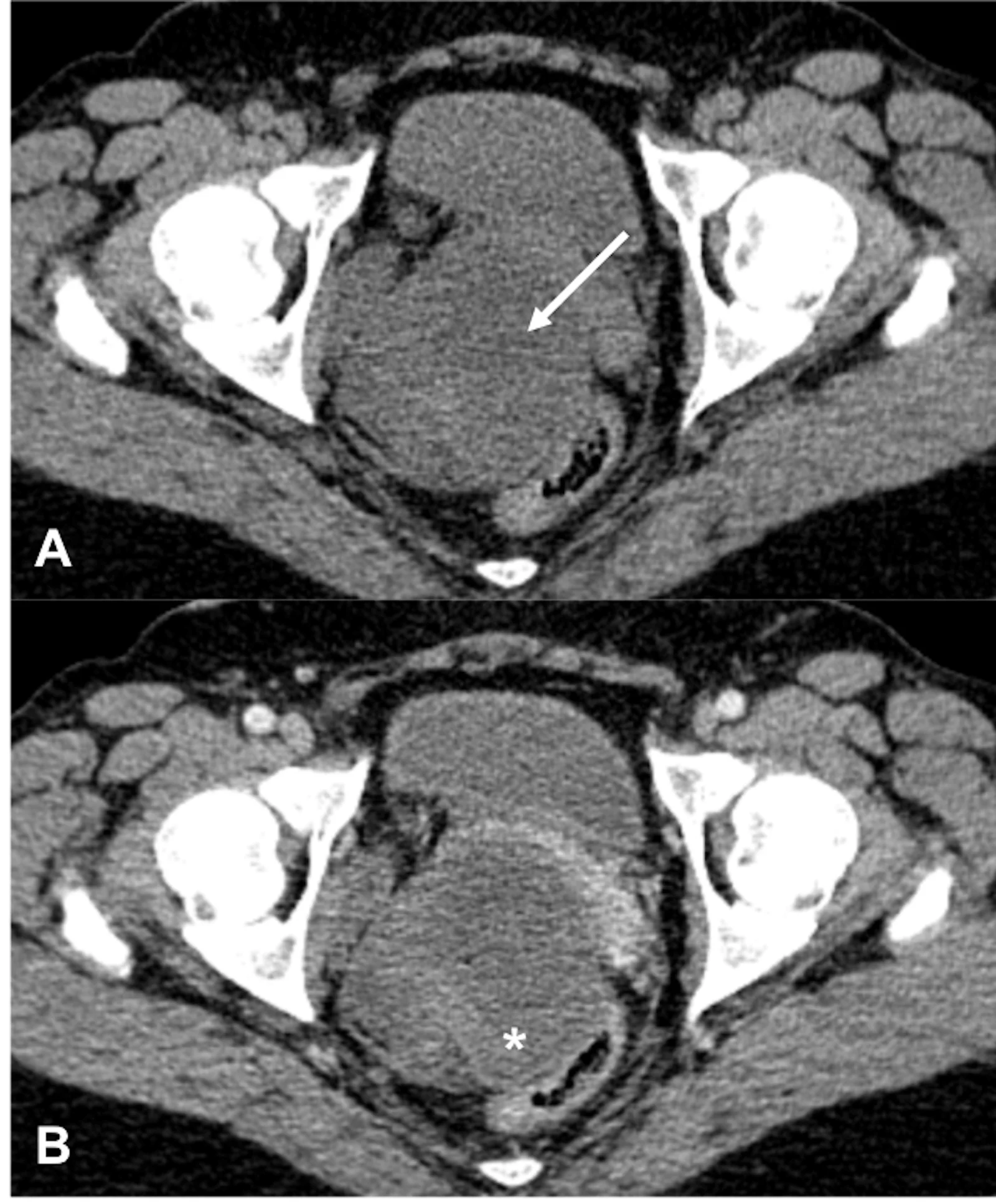
Axial CT images of the pelvic tumor A) Unenhanced CT demonstrates a well-circumscribed hypodense mass centered in the rectovaginal space (arrow), measuring 77 × 69 mm (transverse × anteroposterior). The mass displaces the vagina and urinary bladder anteriorly and the rectosigmoid junction posterolaterally to the left. B) Following intravenous iodinated contrast administration, the mass demonstrates mild heterogeneous enhancement (*).

Computed tomography (CT) demonstrated a well-circumscribed hypodense mass centered in the rectovaginal space. The lesion exerted mass effect on the adjacent pelvic organs. Following intravenous iodinated contrast administration, the mass exhibited mild heterogeneous enhancement (Figure 1).

**Figure 2:**
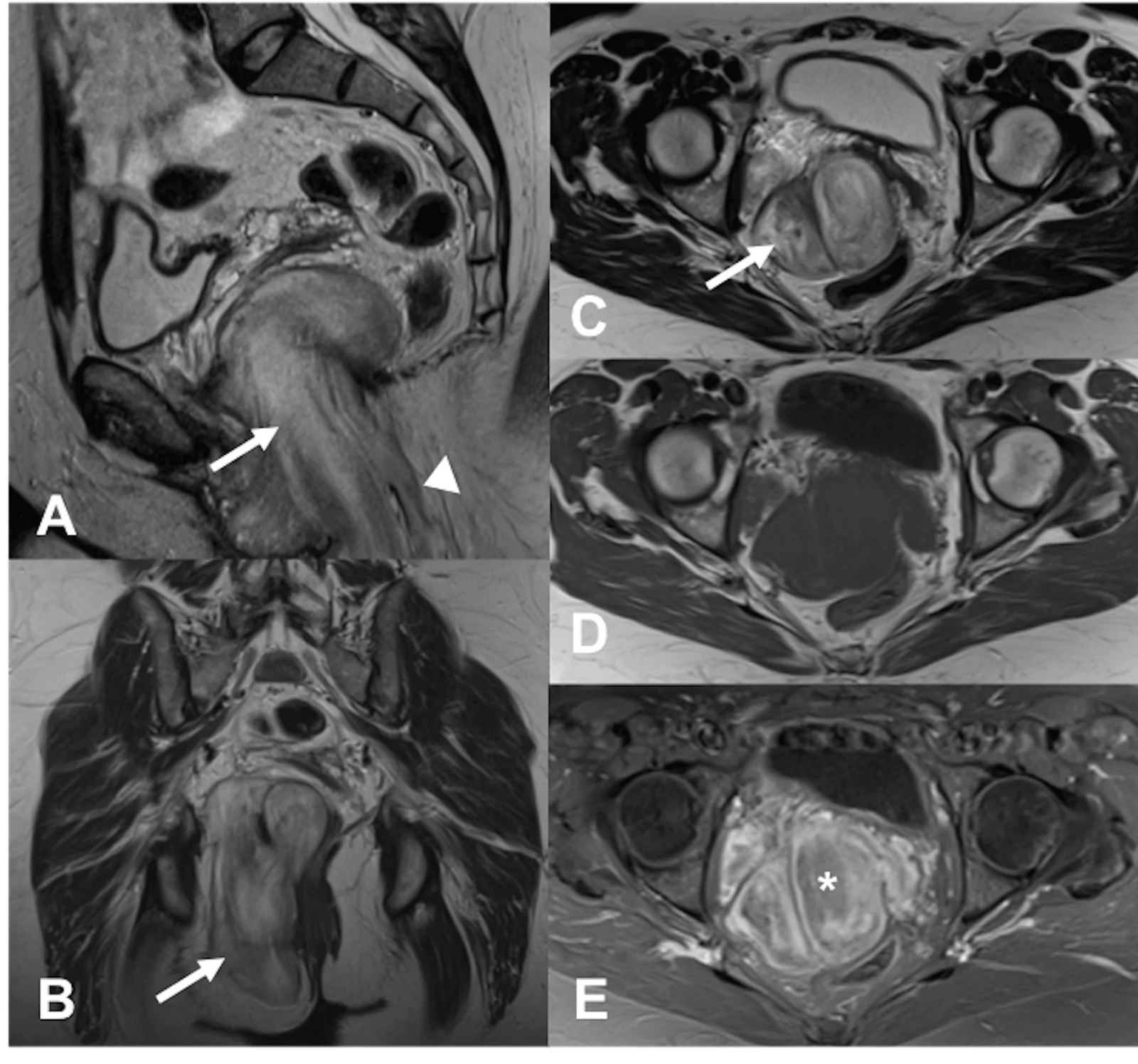
MRI of the pelvic tumor A–C) T2-weighted images in the sagittal (A), coronal (B), and axial (C) planes demonstrate a multilobulated mass centered in the rectovaginal space and right ischiorectal fossa, extending into the intergluteal cleft, with infiltrative finger-like projections into the surrounding soft tissues (arrowhead in A), measuring 93 × 67 × 124 mm (transverse × anteroposterior × craniocaudal). The mass demonstrates high signal intensity with a laminated appearance (arrows). D) Axial unenhanced T1-weighted imaging demonstrates isointense signal relative to skeletal muscle. E) Axial postcontrast fat-suppressed T1-weighted imaging demonstrates intense heterogeneous enhancement with a swirling pattern (*).

Magnetic resonance imaging (MRI) demonstrated a large, multilobulated mass with heterogeneous high signal intensity and a laminated (“swirled”) appearance on T2-weighted images (T2-WI), and isointense signal relative to skeletal muscle on T1-weighted images (T1-WI) (Figure 2). The lesion extended from the right ischiorectal fossa into the intergluteal cleft, with infiltrative finger-like projections into the surrounding soft tissues. Following gadolinium administration, the lesion demonstrated intense heterogeneous enhancement with a swirling pattern. No intralesional calcification, macroscopic fat, hemorrhage, or cystic or necrotic changes were clearly identified on imaging. Considering our patient’s age and premenopausal status, the infiltrative growth pattern, high T2 signal intensity, and laminated (“swirled”) appearance were highly suggestive of pelvic AAM.

**Figure 3:**
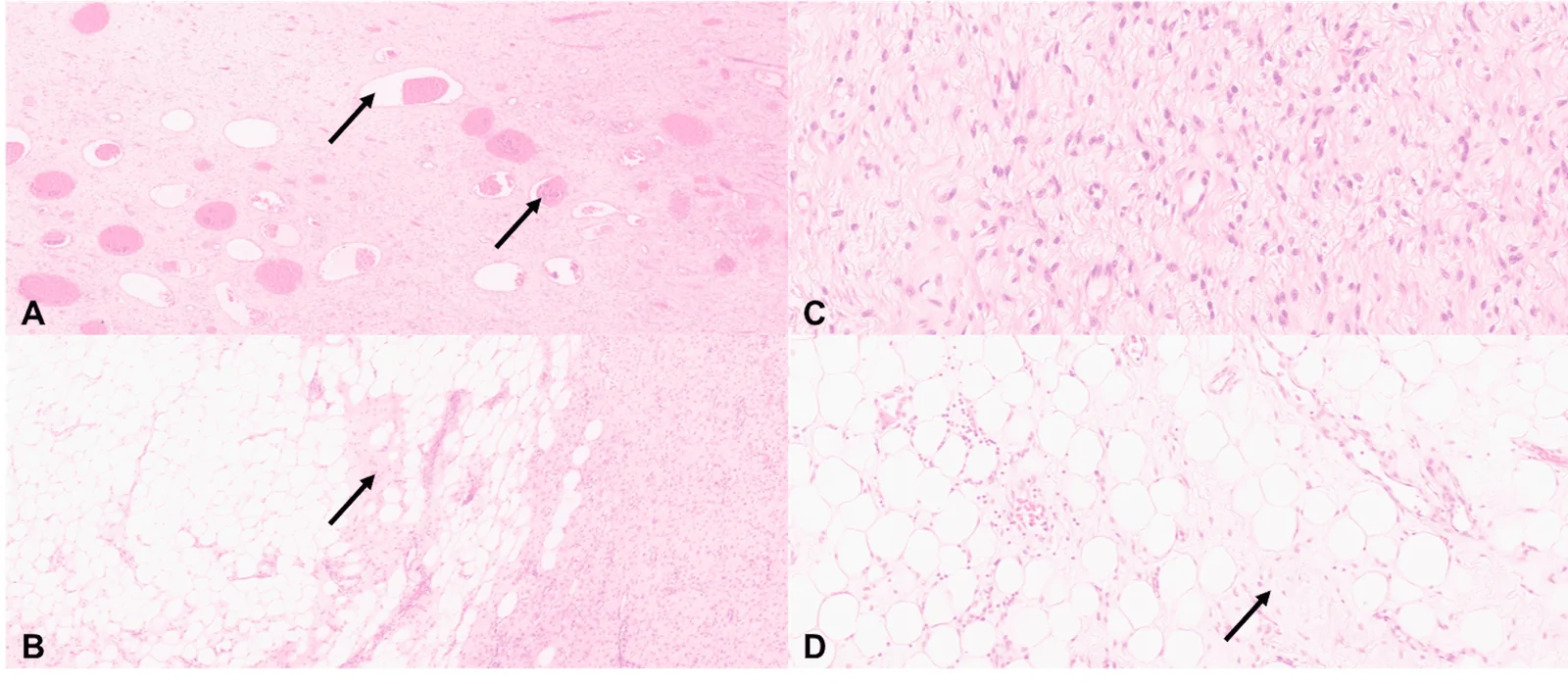
Deep (aggressive) angiomyxoma (Hematoxylin and Eosin - H&E) A) Low-power photomicrograph demonstrating a hypocellular, myxoid lesion composed of spindle and stellate cells within myxoid stroma with scattered delicate collagen fibers. Conspicuous, haphazardly distributed dilated capillaries and larger dilated vessels are present (arrows). There is no evidence of necrosis. B) Intermediate-power view showing the characteristic delicate, thin-walled vasculature and infiltration into adipose tissue (arrow). C) High-power view highlighting bland tumor cells with elongated nuclei, inconspicuous nucleoli, and minimal or absent mitotic activity. D) High-power image of the infiltrative interface, with tumor cells dissecting through adipose tissue, consistent with the locally aggressive behavior of this neoplasm (arrow).

A preoperative CT-guided core needle biopsy was performed at an outside institution, suggesting a mesenchymal tumor. The patient subsequently underwent surgical excision of the lesion with concomitant right oophorectomy, while the left ovary was preserved. Macroscopic residual tumor was present at the resection margins, consistent with an R2 resection. Histopathological analysis of the surgical specimen confirmed the diagnosis of AAM (Figure 3). Immunohistochemical evaluation demonstrated focal positivity for desmin and smooth muscle actin (SMA), positivity for CD34, and strong expression of estrogen and progesterone receptors. Histopathological examination of the right ovary revealed no significant abnormalities.

The postoperative course was uneventful. The presenting symptoms resolved after surgery. Following a multidisciplinary discussion, clinical and imaging surveillance was pursued.

**Figure 4:**
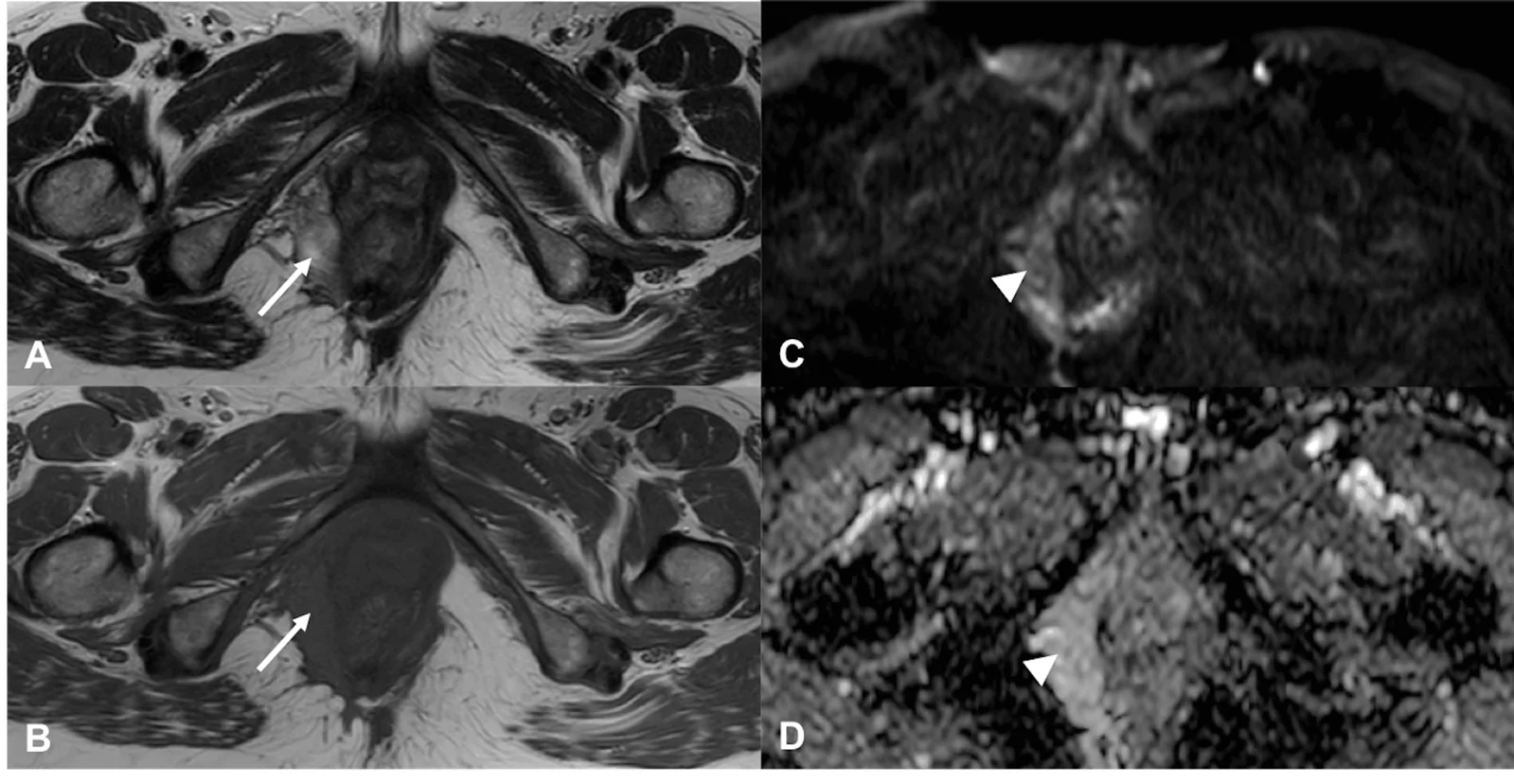
Recurrent tumor at one-year MRI follow-up A, B) Axial T2-weighted (A) and T1-weighted (B) images demonstrate recurrent disease in the right ischiorectal fossa with signal characteristics similar to those of the primary tumor (arrows), measuring 35 × 20 mm (transverse × anteroposterior). C, D) No diffusion restriction is demonstrated on diffusion-weighted imaging (DWI) (C), with a corresponding high apparent diffusion coefficient (ADC) value of 1.78 × 10⁻³ mm²/s on the ADC map (D) (arrowheads).

Follow-up MRI protocol included multiplanar T2-WI, diffusion-weighted imaging (DWI), and apparent diffusion coefficient (ADC) mapping. One year after surgery, MRI demonstrated a recurrent lesion in the right ischiorectal fossa adjacent to postoperative fibrotic changes, with signal characteristics similar to those of the primary tumor (Figure 4). The lesion showed no restricted diffusion and demonstrated a high ADC value of 1.78 × 10⁻³ mm²/s.

**Figure 5:**
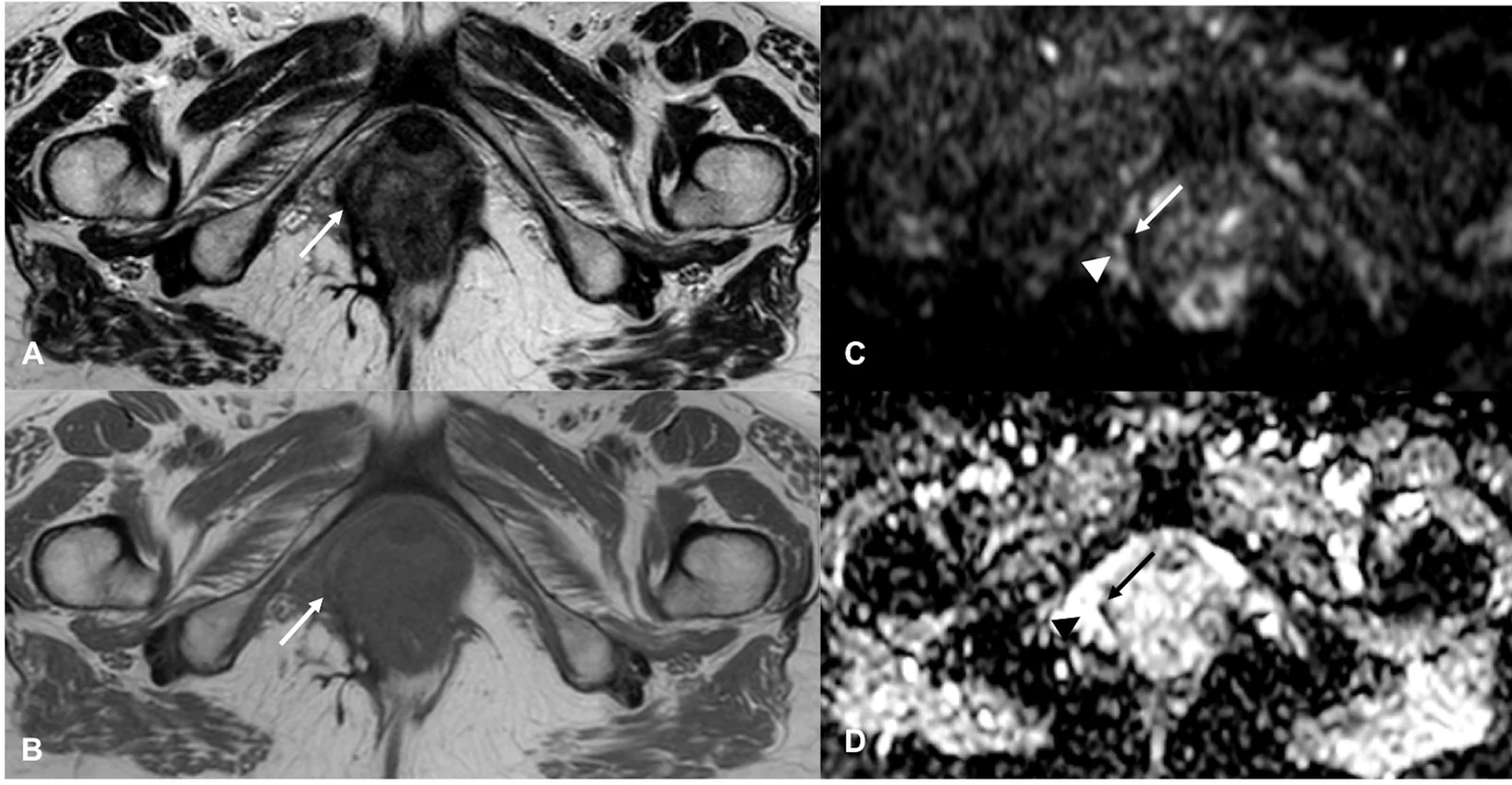
MRI appearance after eight years of continuous gonadotropin-releasing hormone (GnRH) agonist therapy A, B) Axial T2-weighted (A) and T1-weighted (B) images demonstrate complete radiologic tumor regression, with only postoperative fibrotic changes remaining (arrows). C, D) No diffusion restriction is demonstrated on diffusion-weighted imaging (DWI) (arrowhead in C), with corresponding high signal on the apparent diffusion coefficient (ADC) map (arrowhead in D), interspersed with low-signal-intensity fibrotic changes (arrows).

Treatment with a GnRH agonist (goserelin, 3.6 mg monthly) was subsequently initiated. Serial MRI examinations at three-month intervals demonstrated progressive tumor regression, culminating in a complete radiologic response after one year of hormonal therapy. Treatment was well tolerated, with only mild and transient vasomotor symptoms. Thereafter, MRI surveillance was performed annually, confirming complete radiologic remission throughout eight years of continuous GnRH agonist therapy, with no evidence of recurrent disease (Figure 5).

## Discussion

MRI is the preferred imaging modality for diagnosis, assessment of local extent, and follow-up. The characteristic high T2 signal intensity with a laminated or “swirled” appearance represents the key imaging feature of AAM and may also be appreciated on post-contrast sequences. This pattern has been reported in 78-83% of cases and reflects the alternating myxoid and fibrous stromal components [1,2,5]. Additional imaging features include finger-like projections, prominent peripheral vessels, cystic degeneration, and calcifications [1].

Recurrent lesions generally reproduce the signal characteristics of the primary tumor, facilitating their identification during imaging surveillance, as observed in our case [7]. The recurrent lesion demonstrated no restricted diffusion and a high ADC value, in keeping with previously reported findings [1,2,11]. However, DWI was not performed at baseline; therefore, a direct comparison of diffusion characteristics between the primary and recurrent tumors was not possible in the present case.

The differential diagnosis includes other myxoid or myofibroblastic tumors, particularly angiomyofibroblastoma, cellular angiofibroma, pelvic fibromatosis, myxoid leiomyoma, and myxoid liposarcoma. Angiomyofibroblastomas and cellular angiofibromas are generally smaller and more circumscribed than AAM [11,12]. Angiomyofibroblastomas may occasionally contain microscopic fat, detectable on in- and opposed-phase gradient-echo T1-WI [11]. Pelvic fibromatosis generally demonstrates more prominent low-signal-intensity components on T2-WI, whereas a myxoid leiomyoma is typically uterine in origin and may also demonstrate areas of low T2 signal intensity. Myxoid liposarcoma may contain fatty components [1,11]. The presence of a multicompartmental pelvic or perineal mass in a middle-aged woman should raise suspicion for AAM, particularly when demonstrating an insinuative or infiltrative growth pattern, high T2 signal intensity, and a laminated or swirling appearance, as seen in our case [1-3]. Nevertheless, definitive diagnosis relies on histopathological and immunohistochemical evaluation.

Surgical resection remains the primary treatment for AAM; however, complete excision may be challenging because of its infiltrative growth pattern and close relationship with adjacent pelvic structures, potentially resulting in substantial surgical morbidity [1,5]. Importantly, complete resection does not eliminate the risk of recurrence. Similar outcomes have been reported in patients with positive and negative surgical margins, supporting a more conservative surgical approach when complete excision would require extensive surgery, provided that long-term surveillance is maintained [5,11].

Favorable responses to hormonal therapy in AAM have been described in recurrent, residual, or unresectable disease, as well as in the neoadjuvant setting to reduce tumor burden before surgery [7,9,10]. However, evidence regarding long-term efficacy remains limited, while the optimal treatment regimen and duration have not been established. A multicenter retrospective study demonstrated an overall response rate of 62% with first-line hormonal therapy, supporting its role as an effective option for disease control [8]. A recent comprehensive review found treatment durations ranging from three months to five years, with follow-up extending from several months to more than seven years [9]. Furthermore, tumor regrowth or progression after treatment discontinuation has been described, highlighting the uncertainty regarding the durability of response after hormonal therapy withdrawal [7,10]. 

In our patient, GnRH agonist therapy was initiated following MRI-documented recurrence one year after surgery. Follow-up MRI demonstrated progressive tumor regression, culminating in a complete radiologic response after one year of treatment. Complete radiologic remission was subsequently sustained throughout eight years of continuous GnRH agonist therapy, with no imaging evidence of recurrent disease. In the context of the limited long-term data available, this prolonged, longitudinally documented radiologic response highlights both the potential for durable disease control with GnRH agonist therapy and the value of MRI for longitudinal assessment of treatment response.

## Conclusions

MRI plays a central role in the diagnosis, assessment of treatment response, and long-term surveillance of patients with AAM, given the high propensity of this tumor for local recurrence. The frequent expression of estrogen and progesterone receptors provides the biological rationale for hormonal therapy in AAM. In our patient with recurrent pelvic AAM, serial MRI documented complete radiologic remission sustained throughout eight years of continuous GnRH agonist therapy. This uncommon long-term outcome highlights the potential role of hormonal therapy in selected patients as an approach that may reduce the need for extensive repeat surgery.
